# Supplementary material for: The Evolution and Characterization of the RNA Interference Pathways in Lophotrochozoa
Source: Genome Biol Evol. 2024 May 7;16(5):evae098. doi: 10.1093/gbe/evae098 (PMC11114477; doi:10.1093/gbe/evae098)
Supplement: evae098_Supplementary_Data [file evae098_supplementary_data.zip › Supplementary_materials.zip!/selected_miRNAs/NoGe_selected_miRNAs/mirdeep_pdf_files/NMRB01000426.1_Notospermus_geniculatus_strain_Ushimado_scaffold426,_whole_genome_shotgun_sequence_13044.pdf]

Provisional ID	:	NMRB01000426.1_Notospermus_geniculatus_strain_Ushimado_scaffold426,_whole_genome_shotgun_sequence_13044
Score total	:	3547.5
Score for star read(s)	:	3.9
Score for read counts	:	3538.8
Score for mfe	:	1.8
Score for randfold	:	0
Score for cons. seed	:	3
Total read count	:	6953
Mature read count	:	5820
Loop read count	:	0
Star read count	:	1133

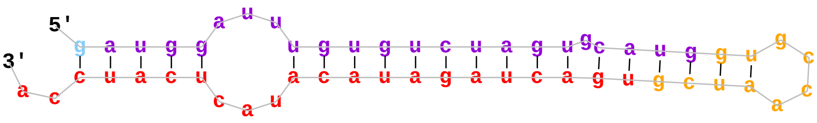

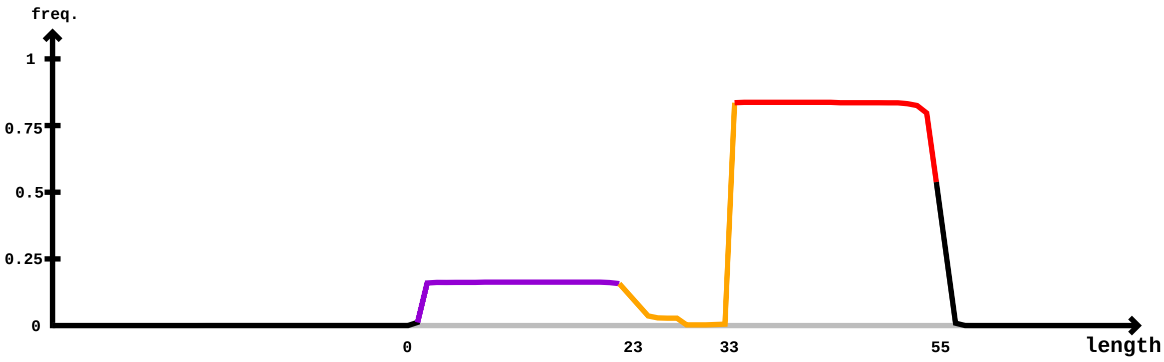


```
                                    Star                          Mature
5'- guuauuggguguucaaacugcagaacaugcucgggugguauggauuugugucuagugcauggugccaaucgugacuagauacauacucauccauauuggcaguuccuucauca  -3'   obs
```

```
5'- guuauugguguucaaacugcagaacaugcucgggugg auggauuugugucuagugcaug gugccaaucg ugacuagauacauacucaucca uauuggcaguuccuucauca  -3'   obs
    guuauugguguucaaacugcagaacaugcucgggug gauggauuugugucuagugcaug gugccaaucg ugacuagauacauacucaucca uauuggcaguuccuucauca        exp
    ......(((......)))...((((.((((...((((((((...((((((((((.((((((....))))))))))))))))...))))))))...))))))))........   reads   mm   sample
    ....................................gauggauuugugucuagugcauggu..................................................   4       0    Fm1
    ....................................gauggauuugugucuagugcauggG..................................................   1       1    Fm1
    .....................................auggauuugugucuagugca......................................................   9       0    Fm1
    .....................................auggauuugugucuagugcau.....................................................   14      0    Fm1
    .....................................auggauuugugucuagugcaug....................................................   95      0    Fm1
    .....................................auggauuugugucuagugcauU....................................................   3       1    Fm1
    .....................................auggauuugugucuagCgcaug....................................................   1       1    Fm1
    .....................................auggauuuguAucuagugcaug....................................................   1       1    Fm1
    .....................................auggauuugugucuagugcaugg...................................................   10      0    Fm1
    .....................................auggauuugugucuagugcauggu..................................................   14      0    Fm1
    .....................................auggauuugugucuagugcauggG..................................................   1       1    Fm1
    .....................................auggauuugugucuagugcauggA..................................................   3       1    Fm1
    .....................................auggauuugugucuaguUcauggu..................................................   1       1    Fm1
    .....................................auggauuugugucuagugcaugguU.................................................   3       1    Fm1
    .....................................auggauuugugucuagugcauggugcc...............................................   29      0    Fm1
    .....................................auggauuugugucuaguUcauggugcc...............................................   1       1    Fm1
    ...........................................uugugucuagugcauggugcc...............................................   6       0    Fm1
    ....................................................................gugacuagauacauacucaucc.....................   1       0    Fm1
    .....................................................................ugacuagauacauacucau.......................   4       0    Fm1
    .....................................................................ugacuagauacGuacucauc......................   1       1    Fm1
    .....................................................................ugacuagauacauacucauc......................   24      0    Fm1
    .....................................................................ugacuagauacauacucaucU.....................   7       1    Fm1
    .....................................................................ugacuGgauacauacucaucc.....................   1       1    Fm1
    .....................................................................ugacuagauacauacucaucc.....................   171     0    Fm1
    .....................................................................ugacuagauacauacucUucc.....................   1       1    Fm1
    .....................................................................ugacuaCauacauacucaucc.....................   1       1    Fm1
    .....................................................................ugacuagauCcauacucaucc.....................   2       1    Fm1
    .....................................................................ugacuagauacaCacucaucc.....................   3       1    Fm1
    .....................................................................ugacuagauacauacucGucc.....................   1       1    Fm1
    .....................................................................ugacuagauaAauacucaucc.....................   1       1    Fm1
    .....................................................................ugacuagauacauacucaucca....................   81      0    Fm1
    .....................................................................ugacuagauacauacucauccG....................   5       1    Fm1
    .....................................................................ugacuagauacauacuAaucca....................   1       1    Fm1
```

```
                                     Star                           Mature
guuauugguguucaaacugcagaacaugcucggguggauggauuugugucuagugcauggugccaaucgugacuagauacauacucauccauauuggcaguuccuucauca
.....................................................................ugacuagauacauacucauccU....................    49    1    Fm1
.....................................................................ugacuagauacauacucauccCu...................    2     1    Fm1
.....................................................................ugacuagauacauacucauccau...................    8     0    Fm1

....................................gauggauuugugucuagugcaGg....................................................    1     1    Fm2
....................................gauggauuugugucuagugcaug....................................................    24    0    Fm2
....................................gauggauuugugucuagCgcauggugcc...............................................    1     1    Fm2
....................................gauggauuugugucuagugcauggugcc...............................................    19    0    Fm2
.....................................auggauuuguAucuagugcaug....................................................    1     1    Fm2
.....................................auggauuugugucuagGgcaug....................................................    1     1    Fm2
.....................................auggauuugugucuaguAcaug....................................................    1     1    Fm2
.....................................auggauuugugucuagugcaug....................................................    188   0    Fm2
.....................................Nuggauuugugucuagugcaug....................................................    1     1    Fm2
.....................................auggauuugugucuaguUcaug....................................................    1     1    Fm2
.....................................auggauuugugucuaguCcaug....................................................    1     1    Fm2
.....................................auggauuugugucuagCgcaug....................................................    1     1    Fm2
.....................................auggauuugugucuagugcauggU..................................................    1     1    Fm2
.....................................auggauuugugucuagugcauggugcc...............................................    28    0    Fm2
.....................................auggauuugugucuagCgcauggugcc...............................................    1     1    Fm2
.....................................auggauuugugucuaguCcauggugcc...............................................    1     1    Fm2
...................................................................cgugacuagauacauacucauc......................    7     0    Fm2
.....................................................................ugacuagauCcauacucau.......................    1     1    Fm2
.....................................................................ugacuagauCcauacucauc......................    1     1    Fm2
.....................................................................ugacuagauacauacucauc......................    14    0    Fm2
.....................................................................ugacuagauacGuacucauc......................    1     1    Fm2
.....................................................................ugGcuagauacauacucaucc.....................    1     1    Fm2
.....................................................................ugacuagauacauaAucaucc.....................    1     1    Fm2
.....................................................................ugacuagauacaCacucaucc.....................    6     1    Fm2
.....................................................................ugacuagauacauacucaucc.....................    148   0    Fm2
.....................................................................ugacuagauacauacucUucc.....................    3     1    Fm2
.....................................................................ugacuagaGacauacucaucc.....................    1     1    Fm2
.....................................................................ugacuagauacauacuUaucc.....................    1     1    Fm2
.....................................................................ugacuagUuacauacucaucc.....................    1     1    Fm2
.....................................................................ugacuagauacauacucauUc.....................    6     1    Fm2
.....................................................................ugacuagauacauacucaucU.....................    6     1    Fm2
.....................................................................Ngacuagauacauacucaucc.....................    1     1    Fm2
.....................................................................ugacuagauacauacuGaucc.....................    1     1    Fm2
.....................................................................ugacuagauCcauacucaucc.....................    8     1    Fm2
.....................................................................ugacuagauacauacucauccU....................    27    1    Fm2
.....................................................................ugacuagauacauacucaucca....................    219   0    Fm2
.....................................................................ugacuaNauacauacucaucca....................    1     1    Fm2
.....................................................................ugacuagauacauacucUucca....................    1     1    Fm2
.....................................................................ugacuagauacauacucCucca....................    2     1    Fm2
.....................................................................ugacuagauacauacucauccG....................    1     1    Fm2
.....................................................................ugacuagauacauacucauccau...................    4     0    Fm2
.....................................................................ugacuagauacauacucauccUu...................    10    1    Fm2
.....................................................................ugacuagauacauacucauccauU..................    8     1    Fm2

.....................................auggauuugugucuagugc.......................................................    11    0    Mm1
.....................................auggauuugugucuaguAcaug....................................................    1     1    Mm1
.....................................auggauuugugucuagugcaug....................................................    26    0    Mm1
.....................................auggauuugugucuagugcauggu..................................................    7     0    Mm1
......................................uggauuugugucuagugcauggugcc...............................................    15    0    Mm1
....................................................................gugacuagauacauacucaucc.....................    2     0    Mm1
.....................................................................ugacuagauacauacuca........................    19    0    Mm1
.....................................................................ugacuagauCcauacucau.......................    2     1    Mm1
.....................................................................ugacuagauacauacucauc......................    3     0    Mm1
.....................................................................ugacuagauCcauacucauc......................    1     1    Mm1
.....................................................................ugacuagauacaCacucauc......................    1     1    Mm1
.....................................................................ugacuagauaAauacucaucc.....................    2     1    Mm1
.....................................................................ugacuagauaGauacucaucc.....................    1     1    Mm1
.....................................................................ugacuagauacauacucCucc.....................    1     1    Mm1
.....................................................................ugacuagauCcauacucaucc.....................    4     1    Mm1
.....................................................................ugacuagauacauacuGaucc.....................    1     1    Mm1
.....................................................................ugacuagauacauacuAaucc.....................    2     1    Mm1
.....................................................................ugacuagauacauacuUaucc.....................    1     1    Mm1
.....................................................................ugacuagauacaCacucaucc.....................    2     1    Mm1
.....................................................................ugacuagauacauacucaucU.....................    6     1    Mm1
.....................................................................ugacuagauacGuacucaucc.....................    1     1    Mm1
.....................................................................ugacuagauacauacucUucc.....................    2     1    Mm1
```

Star Mature

```
guuauugguguucaaacugcagaacaugcucggguggauggauuugugucuagugcauggugccaaucgugacuagauacauacucauccauauuggcaguuccuucauca
.....................................................................ugacuagauacauacucaucc.....................   235   0   Mm1
.....................................................................ugacuagauacauacuAaucca....................   2     1   Mm1
.....................................................................ugacuagauaUauacucaucca....................   1     1   Mm1
.....................................................................ugacuagauacauacucUucca....................   2     1   Mm1
.....................................................................ugacuagauacauacuUaucca....................   2     1   Mm1
.....................................................................ugacuagauacauacuGaucca....................   1     1   Mm1
.....................................................................ugacuagauacauacucaucca....................   256   0   Mm1
.....................................................................ugacuagauacauacucGucca....................   2     1   Mm1
.....................................................................ugacuagauacauacucauccU....................   44    1   Mm1
.....................................................................ugacuagauacauacucauccau...................   11    0   Mm1
.....................................................................ugacuagauacauacucauccUu...................   2     1   Mm1
.....................................................................ugacuagauacauacucauccaA...................   12    1   Mm1
.....................................................................ugacuagauacauacucauccauU..................   8     1   Mm1

..............aacugcagaacaugcucggguggUu........................................................................   1     1   Mm2
....................................gauggauuugugucuagugcaug....................................................   6     0   Mm2
....................................gauggauuugugucuagGgcauggugcc...............................................   1     1   Mm2
....................................gauggauuugugucuagugcauggugcc...............................................   1     0   Mm2
.....................................auggauuugugucuagugca......................................................   1     0   Mm2
.....................................auggauuugugucuagugcau.....................................................   3     0   Mm2
.....................................auggauuugugucuagugcauU....................................................   1     1   Mm2
.....................................auggauuugugucuagugcaug....................................................   12    0   Mm2
.....................................auggauuugugucuagugcaugU...................................................   2     1   Mm2
.....................................auggauuugugucuagugcaugg...................................................   1     0   Mm2
.....................................auggauuugugucuagugcauggu..................................................   5     0   Mm2
.....................................auggauuugugucuagugcauggugcc...............................................   14    0   Mm2
.....................................auggauuugugucCagugcauggugcc...............................................   1     1   Mm2
.....................................auggauuugugucuaguCcauggugcc...............................................   2     1   Mm2
.....................................auggauuugugucuaAugcauggugcc...............................................   1     1   Mm2
........................................gauuugugucuagugcaug....................................................   1     0   Mm2
........................................gauuugugucuagugcauggugccaauc...........................................   1     0   Mm2
...........................................................gugccaaucgugacuagauacauacu..........................   1     0   Mm2
.....................................................................ugacuagauCcauacucau.......................   2     1   Mm2
.....................................................................ugacuagauacauacucauc......................   7     0   Mm2
.....................................................................ugacuagauacauacucUucc.....................   2     1   Mm2
.....................................................................ugacuagauaAauacucaucc.....................   1     1   Mm2
.....................................................................ugacuagauacaCacucaucc.....................   2     1   Mm2
.....................................................................ugacuagauacauacucCucc.....................   1     1   Mm2
.....................................................................ugacuagauCcauacucaucc.....................   2     1   Mm2
.....................................................................ugacuagauaGauacucaucc.....................   2     1   Mm2
.....................................................................ugacuagauacauacucaucU.....................   1     1   Mm2
.....................................................................ugacuagauacauacucaucc.....................   121   0   Mm2
.....................................................................ugacuagauacauacucaucca....................   73    0   Mm2
.....................................................................ugacuagauacauacuAaucca....................   1     1   Mm2
.....................................................................ugacuagauaAauacucaucca....................   1     1   Mm2
.....................................................................ugacuagauacauacucauccG....................   1     1   Mm2
.....................................................................ugacuagauacauacucauccC....................   14    1   Mm2
.....................................................................ugacuagauacauacucauccU....................   32    1   Mm2
.....................................................................ugacuagauacauacucGucca....................   1     1   Mm2
.....................................................................ugacuagauacauacucauccUu...................   1     1   Mm2
.....................................................................ugacuagauacauacucauccau...................   8     0   Mm2
.....................................................................ugacuagauacauacucauccCu...................   3     1   Mm2
.....................................................................ugacuagauacauacucauccauU..................   3     1   Mm2
.....................................................................ugacuagauacauacucauccauCu.................   1     1   Mm2

....................................gauggauuugugucuagugcaug....................................................   20    0   Fm3
.....................................auggauuugugucuagugca......................................................   17    0   Fm3
.....................................auggCuuugugucuagugca......................................................   1     1   Fm3
.....................................Uuggauuugugucuagugcau.....................................................   1     1   Fm3
.....................................auggauuugugucuagugcau.....................................................   32    0   Fm3
.....................................auggauuugugucuaguCcaug....................................................   1     1   Fm3
.....................................auggauuuguAucuagugcaug....................................................   1     1   Fm3
.....................................auggauuugugucuaguAcaug....................................................   6     1   Fm3
.....................................auggauuuAugucuagugcaug....................................................   1     1   Fm3
.....................................auggauuugugucuagCgcaug....................................................   1     1   Fm3
.....................................auggauuugugucuGgugcaug....................................................   2     1   Fm3
.....................................auggauuugugucuagAgcaug....................................................   2     1   Fm3
.....................................auggauuugugucuagugcauU....................................................   12    1   Fm3
.....................................auggauuugugucuagugcaug....................................................   322   0   Fm3
.....................................auggauuugugucuagugcaugg...................................................   29    0   Fm3
```

Star **Mature**

```
guuauugguguucaaacugcagaacaugcucgggugggauggauuugugucuagugcauggugccaaucgugacuagauacauacucauccauauuggcaguuccuucauca
.....................................auggauuugugucuagugcaugU...................................................   16    1    Fm3
.....................................auggauuugugucuagugcauggu..................................................   12    0    Fm3
.....................................auggauuugugucuagCgcauggu..................................................   1     1    Fm3
.....................................auggauuugugucuagugcaugUu..................................................   2     1    Fm3
.....................................auggauuugugucuagugcauggU.................................................   4     1    Fm3
.....................................auggauuugugucuagugcauggugcc...............................................   16    0    Fm3
.....................................auggauuugugucuaguCcauggugcc...............................................   1     1    Fm3
...........................................................gugccaaucgugacuagauac...............................   11    0    Fm3
............................................................ugccaaucgugacuagauacauacu..........................   1     0    Fm3
............................................................ugccaaucgugacuagauacauacucaucU.....................   1     1    Fm3
.............................................................gccaaucgugacuagauacauacucau.......................   2     0    Fm3
...................................................................cgugacuagauacauacucaucU.....................   2     1    Fm3
....................................................................gugacuagauacauacucauc......................   3     0    Fm3
....................................................................Cugacuagauacauacucaucc.....................   3     1    Fm3
....................................................................Cugacuagauacauacucaucca....................   1     1    Fm3
.....................................................................ugacuagauacauacucU........................   2     1    Fm3
.....................................................................ugacuagauacaCacuca........................   1     1    Fm3
.....................................................................ugacuagauacauacuAau.......................   1     1    Fm3
.....................................................................ugacuagauCcauacucau.......................   9     1    Fm3
.....................................................................ugacuagauacaCacucau.......................   3     1    Fm3
.....................................................................ugacuagauacauacucau.......................   23    0    Fm3
.....................................................................ugacuagauacauacucauU......................   1     1    Fm3
.....................................................................ugacuagauaAauacucauc......................   1     1    Fm3
.....................................................................ugacuagauacauacCcauc......................   1     1    Fm3
.....................................................................ugacuagauacaCacucauc......................   1     1    Fm3
.....................................................................ugacuagauacauacucauc......................   104   0    Fm3
.....................................................................ugacuagauacGuacucauc......................   6     1    Fm3
.....................................................................uAacuagauacauacucauc......................   1     1    Fm3
.....................................................................ugacuagauacauacuGaucc.....................   1     1    Fm3
.....................................................................ugacuagauGcauacucaucc.....................   1     1    Fm3
.....................................................................ugacuagauacauacuAaucc.....................   4     1    Fm3
.....................................................................ugacuagauacauacucaucc.....................   693   0    Fm3
.....................................................................ugacuagauacaCacucaucc.....................   24    1    Fm3
.....................................................................ugacuagauacauacuUaucc.....................   2     1    Fm3
.....................................................................ugacuagauacauacucGucc.....................   2     1    Fm3
.....................................................................ugacuagauacauacucUucc.....................   1     1    Fm3
.....................................................................ugacuagauaAauacucaucc.....................   2     1    Fm3
.....................................................................ugacuagauacauacucCucc.....................   2     1    Fm3
.....................................................................ugacuagauCcauacucaucc.....................   16    1    Fm3
.....................................................................ugacuagauacGuacucaucc.....................   2     1    Fm3
.....................................................................ugacuagauUcauacucaucc.....................   1     1    Fm3
.....................................................................ugacuagauacauacucaucU.....................   94    1    Fm3
.....................................................................uCacuagauacauacucaucc.....................   1     1    Fm3
.....................................................................ugacuagauacauacucCucca....................   4     1    Fm3
.....................................................................ugacuagauacGuacucaucca....................   2     1    Fm3
.....................................................................ugacuagauaGauacucaucca....................   1     1    Fm3
.....................................................................ugacuagauacauCcucaucca....................   1     1    Fm3
.....................................................................ugacuagauacauacuUaucca....................   1     1    Fm3
.....................................................................ugacuagauacauacucauccU....................   1202  1    Fm3
.....................................................................ugacuagauacauacucUucca....................   6     1    Fm3
.....................................................................ugacuagauaUauacucaucca....................   1     1    Fm3
.....................................................................ugacuagauacauacucauccC....................   27    1    Fm3
.....................................................................ugacuagauacauacucaucca....................   883   0    Fm3
.....................................................................ugacuagauacauacuAaucca....................   2     1    Fm3
.....................................................................ugacuagauUcauacucaucca....................   1     1    Fm3
.....................................................................Ngacuagauacauacucaucca....................   1     1    Fm3
.....................................................................ugacuagauacauacuGaucca....................   1     1    Fm3
.....................................................................ugacuagauaAauacucaucca....................   3     1    Fm3
.....................................................................ugacuagauacauacucGucca....................   5     1    Fm3
.....................................................................ugacuagauacauacucauccaA...................   19    1    Fm3
.....................................................................uCacuagauacauacucauccau...................   1     1    Fm3
.....................................................................ugacuagauUcauacucauccau...................   1     1    Fm3
.....................................................................ugacuagauacauacucCuccau...................   1     1    Fm3
.....................................................................ugacuagauacauacucauccGu...................   1     1    Fm3
.....................................................................ugacuagauacauacucauccCu...................   54    1    Fm3
.....................................................................ugacuagauacauacucauccau...................   93    0    Fm3
.....................................................................ugacuagauacauacucauccUu...................   24    1    Fm3
.....................................................................ugacuGgauacauacucauccau...................   1     1    Fm3
.....................................................................ugacuagauacauacucauccauU..................   32    1    Fm3
.....................................................................ugacuagauacauacucauccaua..................   1     0    Fm3
```

Star Mature

guuauugguguucaaacugcagaacaugcucgggugg**auggauuugugucuagugcaug**gugccaaucg**ugacuagauacauacucaucca**uauuggcaguuccuucauca			
....gacuagauacauacucaucc.....................	1	0	Fm3
....gacuagauacauacucauccU....................	3	1	Fm3
....gacuagauacauacucauccauU..................	1	1	Fm3
....................................Cauggauuugugucuagugcau...	1	1	Mm3
....................................gauggauuugugucuagugcaug..	1	0	Mm3
....................................gauggauuugugucuagugcauggugcc...	3	0	Mm3
.....................................auggauuugugucuagugcau...	3	0	Mm3
.....................................auggauuugugucuagugcaug..	8	0	Mm3
.....................................auggauuugugucuagugcaugg...	2	0	Mm3
.....................................auggauuugugucuagugcauggu..	2	0	Mm3
.....................................auggauuugugucuagugcauggugcc...	34	0	Mm3
.....................................auggauuugugucuagugcauggugccaauc...	1	0	Mm3
....Cugacuagauacauacucaucc.....................	1	1	Mm3
....ugacuagauacauacuca........................	1	0	Mm3
....ugacuagauCcauacucau.......................	1	1	Mm3
....ugacuagauacauacucauc......................	15	0	Mm3
....ugacuagauacaCacucauc......................	1	1	Mm3
....ugacuagauCcauacucauc......................	1	1	Mm3
....ugacuagauacGuacucauc......................	1	1	Mm3
....ugacuagauacauacuUaucc.....................	1	1	Mm3
....ugacuagauacaCacucaucc.....................	1	1	Mm3
....ugacuagauacauacucaucU.....................	17	1	Mm3
....ugacuagauacauacucaucc.....................	148	0	Mm3
....ugacuagauacauacucGucc.....................	1	1	Mm3
....ugacuagauaGauacucaucc.....................	1	1	Mm3
....ugacuagauCcauacucaucc.....................	14	1	Mm3
....ugacuagauacauacucGucca....................	1	1	Mm3
....ugacuagauacauacucauccU....................	210	1	Mm3
....ugacuagauacauacucauccC....................	15	1	Mm3
....ugacuagauacauacuAaucca....................	2	1	Mm3
....ugacuagauacauacuUaucca....................	1	1	Mm3
....Agacuagauacauacucaucca....................	1	1	Mm3
....ugacuagauacauacucauccG....................	2	1	Mm3
....ugacuagauacauacucUucca....................	2	1	Mm3
....ugacuagauacauacuGaucca....................	1	1	Mm3
....ugacuagauacauacucaucca....................	185	0	Mm3
....ugacuagauacauacucauccau...................	12	0	Mm3
....ugacuagauacauacucauccaC...................	1	1	Mm3
....ugacuagauacauacucauccaA...................	2	1	Mm3
....ugacuagauacauacuAauccau...................	1	1	Mm3
....ugacuagauacauacucauccUu...................	6	1	Mm3
....ugacuagauaUauacucauccau...................	1	1	Mm3
....ugacuagauacauacuUauccau...................	1	1	Mm3
....ugacuagauacauacucauccCu...................	21	1	Mm3
....ugacuagauacauacucauccaAa..................	1	1	Mm3
....ugacuagauacauacucauccauU..................	8	1	Mm3
....gacuagauacauacucauc......................	1	0	Mm3
....gacuagauacaCacucaucc.....................	1	1	Mm3
....gacuagauacauacucauccC....................	1	1	Mm3
....gacuagauacauacucaucca....................	2	0	Mm3
